# Carbonservation with Demonstrated Biodiversity and Carbon Gains: Carbon Can Pay But Biodiversity Must Lead

**DOI:** 10.1007/s00267-023-01928-4

**Published:** 2024-01-09

**Authors:** Anthelia J. Bond, Patrick J. O’Connor, Timothy R. Cavagnaro

**Affiliations:** 1 The Waite Research Institute, and The School of Agriculture, Food and Wine, The University of Adelaide, The Waite Campus, PMB 1 Glen Osmond, Adelaide, SA 5064 Australia; 2 The Centre for Global Food and Resources, The University of Adelaide, Adelaide, SA 5005 Australia; 3 The Environment Institute, The University of Adelaide, Adelaide, SA 5005 Australia; 4 College of Science and Engineering, and Office of Graduate Research, Deputy Vice-Chancellor (Research) Portfolio, Flinders University, Bedford Park, SA 5042 Australia

**Keywords:** Carbon sequestration, Biodiversity conservation, Co-benefit markets, Assisted natural regeneration, Temperate woodland

## Abstract

Land use has a critical role to play in both climate change mitigation and biodiversity conservation, and increasingly there have been calls to integrate policies for concurrently meeting Paris Agreement commitments and the UN decade on ecosystem restoration 2021–2030. Currently however, investment activities have been dominated by climate change mitigation activities, including through the development of carbon markets (both voluntary and compliance markets). Whilst climate change mitigation is to be welcomed, the prioritization of carbon in avoided deforestation and reforestation can lead to suboptimal or negative outcomes for biodiversity. Restoration of degraded native vegetation may provide an opportunity for concurrent production of both carbon and biodiversity benefits, by harnessing existing carbon markets without the need to trade-off biodiversity outcomes. Here we demonstrate that carbon sequestered by restoring degraded temperate woodland can pay the price of the restored biodiversity. This is shown using conservative carbon prices in an established market (during both a voluntary and compliance market phase), and the restoration price revealed by a 10-year conservation incentive payment scheme. When recovery rates are high, market prices for carbon could pay the full price of restoration, with additional independent investment needed in cases where recovery trajectories are slower. Using carbon markets to fund restoration of degraded native vegetation thereby provides a solution for constrained resources and problematic trade-offs between carbon and biodiversity outcomes. Multi-attribute markets offer the potential to greatly increase the extent of restoration for biodiversity conservation, while providing an affordable source of carbon sequestration and enhancing economic benefits to landowners.

## Introduction

Climate change and biodiversity loss are arguably the greatest environmental challenges currently facing humanity (Intergovernmental Panel on Climate Change 2022; United Nations 2019). Land use has a critical role to play in both climate change mitigation (Griscom et al. 2017) and biodiversity conservation (Kehoe et al. 2017), however there are competing demands on land for the provision of these and other ecosystem services (Smith et al. 2014). Historically both carbon sequestration and biodiversity conservation increases have been constrained by lack of investment, as the associated ecosystem services have global public goods characteristics (i.e. they are non-excludable and non-rivalrous). The establishment of voluntary and compliance carbon markets in recent years is increasingly providing investment for carbon emissions abatement including land based carbon sequestration (World Bank 2022b), however there are few examples of similar regulatory mechanisms for biodiversity conservation (Madsen et al. 2010), despite large shortfalls in conservation funding (Waldron et al. 2017).

Whilst climate change mitigation is to be welcomed, when land-based interventions for constraining total carbon emissions, such as avoided deforestation and reforestation, are optimized for carbon outcomes, biodiversity outcomes may be suboptimal or even negative (Ferreira et al. 2018; Lindenmayer et al. 2012a; Venter et al. 2009). For example, Venter et al. (2009) found that prioritizing carbon outcomes in avoided deforestation investment would yield much lower biodiversity benefits than the same investment could if biodiversity benefits were prioritized. Similarly, where carbon outcomes are prioritized in reforestation, planting location, species selection, plant density and planting configuration may provide suboptimal biodiversity benefits (Carswell et al. 2015; Paul et al. 2016b), or may even have negative impacts such as facilitating the spread of invasive species or other disruption of ecological processes (Lindenmayer et al. 2012b). Furthermore, concerns have been raised about the certainty of biodiversity benefits from plantings (Collard et al. 2020; Maron et al. 2012) and the timescales required for such benefits to be realized (Munro et al. 2011). Unsurprisingly there have been increasing calls to integrate policies for concurrently addressing the biodiversity and climate change crises (Bush and Doyon 2021; Pettorelli et al. 2021; Pörtner et al. 2021).

Suggested approaches to improving biodiversity outcomes from avoided deforestation and carbon plantings include planning to optimize carbon and biodiversity outcomes (Di Marco et al. 2018; Ferreira et al. 2018; Phelps et al. 2012; Standish and Prober 2020) and co-benefit markets or additional investment streams to purchase biodiversity outcomes (Bryan et al. 2014; Phelps et al. 2012; Rooney and Paul 2017; Summers et al. 2021). However, planning to optimize carbon and biodiversity benefits, without additional investment or markets for biodiversity, may result in trade-offs for carbon outcomes (Anderson-Teixeira 2018; Choi et al. 2022). Furthermore, in the absence of strong regulatory or market mechanisms specifically for biodiversity, biodiversity outcomes are unlikely to be prioritized in forest-sector emissions reduction interventions.

In contrast to avoided deforestation and carbon planting, restoration of degraded or recently cleared native vegetation (commonly referred to as Assisted Natural Regeneration or ANR) could offer a way to optimize biodiversity outcomes from investment in carbon emissions reduction, without the potential trade-offs between biodiversity and carbon outcomes. While avoided deforestation retains existing vegetation and carbon planting establishes vegetation that may or may not naturally occur in the location, ANR facilitates natural regeneration of vegetation by managing threats such as grazing pressure, invasive species and firewood collection. In some cases ANR may also include supplementary planting. Globally, ANR is increasingly being highlighted as a cost-effective strategy for achieving carbon sequestration across large scales whilst also delivering many important biodiversity and other co-benefits (Crouzeilles et al. 2020; Crouzeilles et al. 2017; Dwyer et al. 2009; Evans 2018; Evans et al. 2015; Gilroy et al. 2014; Yang et al. 2018). However, to date these studies have focused on restoration of vegetation with a recent history of clearance, while far less attention has been given to restoration of degraded native vegetation (vegetation with no recent history of clearance but degraded due to weed invasion, overgrazing, and / or other disturbances), despite its critical importance for biodiversity conservation (IPBES 2019; Murphy and van Leeuwen 2021). Furthermore, studies of synergistic carbon and biodiversity outcomes from ANR have been limited to the use of estimated costs for restoration and, to our knowledge, no study to date includes prices from operating markets for biodiversity and carbon to demonstrate this possibility.

Here we investigate whether carbon markets could pay for biodiversity conservation using an empirical case study: a 10-year conservation incentive payment scheme, with quantified biodiversity benefits from restoration of degraded native vegetation (see Bond et al. 2019b), the restoration price revealed through a reverse auction, and located in a peri-urban biodiversity hotspot in temperate southern Australia (Guerin et al. 2016). We use posted carbon prices from Australia’s Emissions Reduction Fund (ERF) (a voluntary market), and its antecedent program (a compliance market). At the conclusion of the 10-year incentive scheme (2015/16), Australia’s ERF accounted for the majority of the world’s traded forest-based emissions reductions (Hamrick and Gallant 2017). Using Australia’s national carbon accounting model FullCAM (Richards and Evans 2004), we model carbon sequestered through restoration of degraded native vegetation under the 10-year incentive contracts. We compare the value of the carbon sequestered by restoration of degraded native vegetation to the average price of restoration revealed through the incentive scheme’s reverse auction.

## Material and Methods

### The Conservation Incentive Payment Scheme

This study uses a conservation incentive payment scheme, Eastern Mt Lofty Ranges BushBids, as a case study. Briefly, this scheme invited private landholders to tender a price for 10-year contracts for restoration of degraded native vegetation. Contracts were established in 2006 and 2007 and management actions included retention of fallen logs, exclusion or management of domestic stock grazing, weed control, and control of grazing pressure from feral and over-abundant native animals, that were additional to landholders’ existing obligations under relevant laws. Restoration actions including grazing pressure management (Daryanto et al. 2013; Paul and Roxburgh 2020; Witt et al. 2011), weed control (Mostert et al. 2017; Shields et al. 2015), and preventing fire wood collection (Macdonald et al. 2015) can support the maintenance and sequestration of carbon in vegetation, debris and soils (Paul et al. 2016a). No markets for carbon sequestration from land management were available to these landholders at the time of price-setting. For further details of the BushBids scheme see (Bond et al. 2018) and (Bond et al. 2019b). The average price of the restoration contracts was AUD$59 ha^−1^ yr^−1^ (O’Connor et al. 2008).

### Study Area and Sites

The study area is within the eastern Mt Lofty Ranges of South Australia, a recognized centre of plant biodiversity (Guerin et al. 2016). It has a temperate climate with a wide ranging annual average rainfall between approximately 290 mm in the north east and approximately 890 mm in the south west (Bureau of Meteorology 2014). The area’s native vegetation mainly consists of eucalypt dominated forests and woodlands and has been reduced to less than 10% of its former extent (Department of Environment Water and Natural Resources 2011). We modeled carbon sequestration at twelve woodland sites contracted through the BushBids scheme. These sites contained a variety of woodland types which are recognized priorities for immediate conservation (Bergstrom et al. 2021; Prober et al. 2005).

### FullCAM Model and Emission Reduction Fund (ERF) Methodology

To estimate carbon sequestration through restoration of degraded native vegetation, we used the FullCAM model, version 4.0.3.26 (Richards and Evans 2004) which was developed by the Australian Government, for national carbon accounting. Further documentation about FullCAM is available online (Australian Government 2023b). FullCAM models tree growth, litter decomposition and changes in soil carbon in relation to fire, harvest, cropping, grazing and spatially linked productivity information (Richards and Evans 2004). At the time of this study, no methodologies for modeling carbon sequestration from management or restoration of degraded native woodlands with FullCAM had been approved under the ERF. We therefore designed a modeling procedure (outlined below) following relevant components of approved methodologies “Reforestation by Environmental or Mallee Plantings - FullCAM” and “Human-induced regeneration of a permanent even-aged native forest 1.1” (Department of the Environment and Energy 2016a, b, 2018).

### Model Settings and Scenarios

Carbon sequestration from restoration was estimated by subtracting modeled carbon stocks under a business-as-usual scenario from modeled carbon stocks under a paired restoration scenario at the conclusion of a 10-year restoration period (2006–2016). We estimated carbon sequestration in this manner under nine scenario pairs with varying vegetation age and ecosystem degradation rate. Each of the nine scenario pairs included one of three fire events aligned with major historical wildfires in the study environment and one of three ecosystem degradation rates (Table 1). These ecosystem degradation rates were used to represent the effects (loss of carbon from vegetation and debris) of degrading processes such as grazing pressure from stock, feral animals and over-abundant native animals as well as weed invasion and firewood collection. Vegetation age since fire and ecosystem degradation rate were key, user-defined model settings influencing modeled recovery rates. In this study we use the term recovery rate to refer to the rate of increase in carbon in vegetation and debris, not to recovery of biodiversity.

**Table 1:** Modeled scenarios with year of last fire and degradation rate

		Degradation rate (proportion of default growth rate)		
		high (0.25)	medium (0.5)	low (0.75)
Year of last fire (year)	1983	1983, high	1983, medium	1983, low
	1955	1955, high	1955, medium	1955, low
	1939	1939, high	1939, medium	1939, low

The study landscape and its temperate woodlands are relatively fire prone with fire frequency estimated to be multi-decadal (Bradstock 2010; Hobbs 2002). In each scenario we used one of three historic major wildfire events; 1983, 1955 and 1939 (Healey 1985) to represent the range in vegetation age since fire in the study area (Table 1). We used the FullCAM model event type “Wildfire-trees killed” affecting 100% of the site. Parameter values for this event type in the FullCAM model included 100% of stems killed, with 10% combustion to the atmosphere, and 90% stem loss to deadwood pools (Surawski et al. 2012).

The forest treatment “age advance” was used to model ecosystem degradation and was effectively a discounted growth rate, representing the carbon accumulation inhibiting impacts of grazing pressure, weed invasion and firewood collection (see Table 1). Discounted growth rates were initiated in 1946, in line with post-World War II agricultural intensification in southern Australia (Duncan and Dorrough 2009) for the earliest fire scenario, and three years after fire in the more recent fire scenarios. We selected three plausible degradation levels including growth setback of; 3 in 4 years (0.25 times default growth rate), 1 in 2 years (0.5 times default growth rate), and 1 in 4 years (0.75 times default growth rate).

**Fig. 1:**
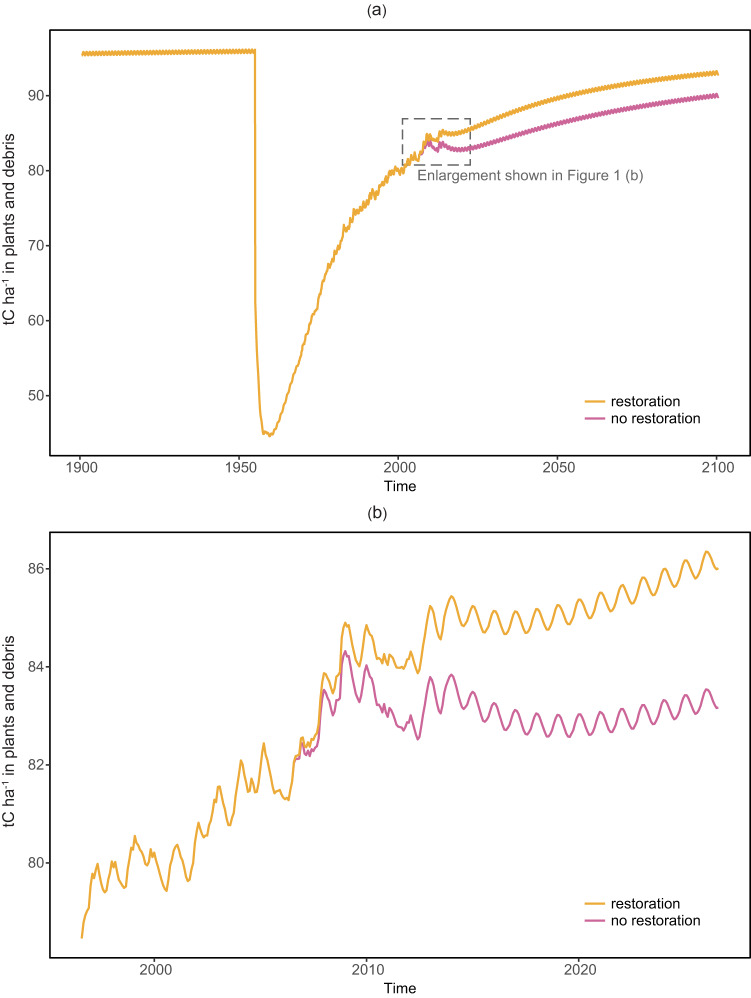
Modeled carbon in plants and debris at a typical study site, with and without restoration beginning in 2006, under the 1955 fire, medium degradation scenario. Time period 1900–2030 shown at **a**, time period 1996–2026 shown at **b**

All simulations were initiated in the year 1606 to allow a period of more than 300 years for stabilization of carbon stocks prior to modeled events including fire, degradation and restoration (Fig. 1a). In 2006, at the start of the restoration period, degradation was removed to simulate the mitigating effects of restoration (Fig. 1b). Estimated 2016 carbon stocks were then compared to the paired and otherwise identical scenario where degradation continued, to provide an estimate of the difference in carbon sequestered over the 10-year period. We used the 10-year difference in carbon sequestered between the restoration and business as usual scenarios to calculate the average difference in carbon sequestered per hectare, per year, i.e. the additional carbon sequestered. The average additional carbon (ha^−1^ yr^−1^) sequestrated by restoration was then multiplied by the carbon price and compared with the restoration price ($59 ha^−1^ yr^−1^) from the conservation incentive payment scheme.

We used the “Mixed species environmental planting–temperate” model within FullCAM, with a geometric block planting of 500–1500 plants per ha with trees making up at least 75% of plants. FullCAM’s “mixed species environmental planting-temperate” model was recently refined and calibrated (Paul et al. 2013, 2015), and was considered to be the most suitable for this study in the absence of appropriate, calibrated models specifically for degraded native woodland in the study area.

### Data Analysis and Presentation

To calculate the additional carbon sequestered from restoration, we performed the following operations. At the end of the 10-year restoration period (30 June 2016), the carbon stocks in the business as usual scenario were subtracted from the carbon stocks in the restoration scenario for each scenario pair. The additional carbon was converted from tC ha^−1^ to tCO_2_e ha^−1^ by multiplying by 44 and dividing by 12. Following conversion to tCO_2_e ha^−1^, we divided the additional carbon by 10 to give tCO_2_e ha^−1^ yr^−1^. Mean and standard deviation were calculated for each scenario from the 12 sample sites. We plotted the relationship between carbon price and the per cent of the restoration price that could be covered by the carbon sequestered using a line for each scenario where the slope of the line is the additional carbon sequestered (tCO_2_e ha^−1^ yr^−1^) divided by the restoration price ($59 ha^−1^ yr^−1^), multiplied by 100.

Analysis was performed in R (R Core Team 2017) and plots created with the *ggplot2* package (Wickham 2009). Data generated for the study and R code used for analysis are available in the Figshare repository 10.25909/5cf08c6820044 (Bond et al. 2019a). To protect the privacy of conservation incentive payment scheme participants, spatial location of study sites has been withheld.

## Results and Discussion

### Carbon Can Pay for Biodiversity Conservation

**Fig. 2:**
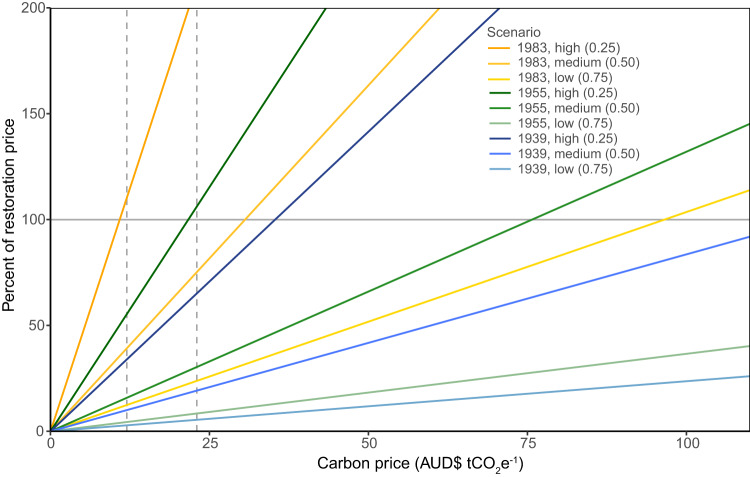
Percent of restoration price covered by carbon sequestered under modeled scenarios (*n* = 12). Scenario parameters are year of last fire (1983, 1955, 1939) and degradation rate (high, medium, low (proportion of default growth rate 0.25, 0.5, 0.75)). Conservative carbon prices in the Australian market, during the restoration time period (2006–2016), AUD$12 tCO_2_e^−1^ and AUD$23 tCO_2_e^−1^, are indicated with dashed lines

We found that sequestered carbon could pay all, or a substantial proportion, of the price of restoring degraded native vegetation (the price revealed by the conservation incentive payment scheme) under plausible scenarios of vegetation age and degradation rate, with carbon prices posted in Australia’s carbon market (Fig. 2). With a carbon price of AUD$23 tCO_2_e^−1^ the full price of restoration was covered by carbon sequestration alone when recovery rates were high (e.g. scenarios with 1983 or 1955 fire/clearance and high degradation rate). Similarly, with a carbon price of AUD$12 tCO_2_e^−1^ the full price of restoration was covered by carbon sequestration under the scenario with the highest recovery rate (1983 fire/clearance and high degradation rate). In other scenarios, at these conservative carbon prices, carbon sequestration could pay a substantial proportion of the restoration price, but co-investment or market-differentiation for the biodiversity co-benefits would have also been required.

This shows for the first time that the price of restoring native vegetation for biodiversity conservation could be covered by trading concurrently produced carbon co-benefits, using restoration prices from an operating conservation incentive payment scheme where biodiversity outcomes are known (Bond et al. 2019b). Linking biodiversity and carbon markets in this way has the potential to improve biodiversity benefits from forest-based carbon sequestration investment without the trade-offs that are likely where carbon markets are well established and biodiversity markets are missing, or prices are not calibrated to demonstrated biodiversity outcomes. Rather than seeking biodiversity co-benefits from investments optimized for carbon markets, biodiversity priorities could be directly targeted (e.g. species, ecosystems, landscapes), with full or partial subsidization from trading carbon as a co-benefit of biodiversity conservation; ensuring the biodiversity conservation ‘dog’ is wagging the carbon sequestration ‘tail’, and potentially extending the reach of constrained budgets for biodiversity conservation. However, careful policy design will be required to minimize transaction costs and overcome other challenges presented by asynchronous carbon and biodiversity markets and policies (Summers et al. 2019).

### Influence of Recovery Rates

We used time since fire (or clearance), and degradation rate to model a range of vegetation recovery rates. In this study we use the term recovery rate to refer to the rate of increase of carbon in vegetation and debris, rather than biodiversity recovery. As expected, younger vegetation with a higher growth rate, and therefore a higher recovery rate, provided more sequestered carbon over the 10-year restoration period. Because we assumed degrading processes were completely negated by restoration, higher rates of degradation also provided higher recovery rates in this study. Average rates of carbon sequestration estimated ranged from less than 0.1 tC ha^−1^ yr^−1^ to 1.5 tC ha^−1^ yr^−1^ (Table 2). The mid-point of this range falls within a previously estimated range for carbon sequestration potential in temperate Australian woodlands (0.35–0.77 tC ha^−1^ yr^−1^) (Paul et al. 2016a). Degradation and recovery are likely to depend on fire and management history in addition to site productivity (Paul et al. 2016a). Site productivity is already accounted for in FullCAM models and there is some existing capability to account for fire and management history that could be further refined. Additionally, strategies to account for fire and management history will be required in policies and standardized procedures for estimating carbon sequestration from degraded native vegetation restoration.

**Table 2:** Mean ± standard deviation for additional carbon sequestered under the nine scenarios (*n* = 12)

		Degradation rate (proportion of default growth rate)					
		high (0.25)		medium (0.5)		low (0.75)	
		tC ha^−1^ yr^−1^	tCO_2_e ha^−1^ yr^−1^	tC ha^−1^ yr^−1^	tCO_2_e ha^−1^ yr^−1^	tC ha^−1^ yr^−1^	tCO_2_e ha^−1^ yr^−1^
Year of last fire (year)	1983	1.48 ± 0.54	5.42 ± 1.98	0.53 ± 0.19	1.93 ± 0.71	0.17 ± 0.06	0.61 ± 0.22
	1955	0.74 ± 0.27	2.72 ± 1.00	0.21 ± 0.08	0.78 ± 0.28	0.06 ± 0.02	0.22 ± 0.08
	1939	0.46 ± 0.17	1.67 ± 0.61	0.13 ± 0.05	0.49 ± 0.18	0.04 ± 0.01	0.14 ± 0.05

The benefit of linking carbon and biodiversity markets can be realized where carbon and biodiversity recovery rates have a positive (but not necessarily linear) relationship. We acknowledge that restoration of degraded native vegetation will not produce carbon benefits if restoration transitions vegetation from higher to lower carbon stocks (e.g. forest to grassland). The relationship between recovery rates for carbon and biodiversity is not yet well understood for many systems or across recovery trajectories (e.g. Fu et al. 2017; Guo et al. 2019; Matos et al. 2020). Further research on these relationships may enable more precise targeting of restoration policies and programs.

### Influence of Carbon Price

Carbon price has a large influence on the proportion of restoration price that can be covered by carbon markets. Here we used two prices for carbon based on markets operating during the 10-year conservation incentive scheme. The lower of the two, (AUD$12 tCO_2_e^−1^) is the average price paid by the ERF in the 11 auctions which purchased 215 Mt CO_2_e between 2015 and 2020 inclusive (Australian Government 2023a). This is a conservative price, driven by the ERF policy principle to purchase the lowest cost carbon abatement (Australian Government 2014). In practice these emissions reductions have largely been from two vegetation methods; avoided deforestation and assisted natural regeneration (ANR) on marginal land requiring little management intervention to assist regeneration (Burke 2016; Evans 2018). Both carbon prices used in this study are at the lower end of the global range of carbon prices (World Bank 2022a) and are well below the estimated median social cost of carbon emissions (US$400 tCO_2_e^−1^) (Ricke et al. 2018). Since the conclusion of the case study conservation incentive payment scheme, the spot price of carbon in the Australian voluntary market peaked at just above AUD$57 tCO_2_e^−1^ in January 2022 (Jarden 2022) and the most recent ERF auction had an average price of AUD$17.12 tCO_2_e^−1^ (Australian Government 2023a). Furthermore, at the conservative price of AUD$12-17 tCO_2_e^−1^, supply of carbon sequestration through carbon plantings has been very limited in southern Australia, with estimates showing that prices of at least AUD$38 tCO_2_e^−1^ would be required to increase supply (Bryan et al. 2014; Regan et al. 2020).

### Model Caveats

The modeled carbon shown here includes only carbon in plants (above and below-ground biomass) and debris. The inclusion of soil carbon pools may increase estimates, especially over longer time periods (Paul et al. 2016a, 2018). We used the FullCAM model, which is employed by approved methodologies for assessing carbon credits under the regulated ERF market, however no methodology for restoration of degraded native vegetation had been approved at the time of writing. In the absence of a FullCAM model specific to the study system we used one that was considered to provide a realistic substitute (please refer to sections FullCAM Model and Emission Reduction Fund (ERF) Methodology and Model Settings and Scenarios for details). Further refinement and calibration of modeling and methodologies may therefore improve the accuracy of carbon sequestration estimates.

## Conclusions

We have shown here that carbon sequestration can pay for restoration of degraded temperate woodlands using a real, revealed-price conservation incentive payment scheme, under conservative carbon prices with plausible ecosystem degradation and recovery rates. This presents an opportunity to increase the extent of restoration within constrained budgets for biodiversity conservation. It also offers an affordable source of carbon sequestration with demonstrated biodiversity benefits. The use of carbon markets to fund restoration of degraded native vegetation thereby provides a means to give biodiversity outcomes precedence in forest-based carbon emissions reduction, and overcome challenges posed by constrained resources for addressing the duel intersecting problems of climate change and biodiversity loss. To enable trading of carbon sequestered through restoration, suitable regulatory and policy conditions are required, including regulatory frameworks for carbon markets and methodologies to calculate carbon sequestrated by restoration of degraded native vegetation.
